# Ethical perspectives of obstetricians and gynecologists on induced abortion and conscientious objection in Türkiye: a phenomenological study

**DOI:** 10.1186/s12910-026-01380-z

**Published:** 2026-02-26

**Authors:** Maide Barış, Hatice Büşra Arısın, Kerem Arısın, Sare Dilara Koltuk, Aliye Çağlar, Aleyna Nur Ortaklar, Berkay Pehlivaner, Merve Saraçoğlu, Julian Savulescu

**Affiliations:** 1 Department of Medical History and Ethics, Marmara University, Faculty of Medicine, Başıbüyük Campus, No:9/2, Office: 3065, Maltepe, Istanbul, 34854 Türkiye; 2 Department of Public Health, Marmara University, Faculty of Medicine, Başıbüyük Campus, No:9/2, Maltepe, Istanbul, 34854 Türkiye; 3 Marmara University, School of Medicine, Başıbüyük Campus, No:9/2, Maltepe, Istanbul, 34854 Türkiye; 4 Department of Medical Education, Recep Tayyip Erdoğan University, Faculty of Medicine, Rize, Türkiye; 5 Centre for Biomedical Ethics, Yong Loo Lin School of Medicine, National University of Singapore, Singapore, Singapore; 6 Uehiro Oxford Institute, University of Oxford, Oxford, UK

**Keywords:** Induced abortion, Conscientious objection, Clinical ethics, Reproductive rights, Qualitative research

## Abstract

**Background:**

Although abortion has been legally permitted in Türkiye for up to ten weeks since 1983, access in public hospitals has been reported to decline over the past decade due to clinicians’ refusals and institutional restrictions, despite the absence of a formal legal framework for conscientious objection. This study aims to explore the experiences and ethical reasoning of Turkish obstetricians and gynecologists regarding abortion practice and conscientious objection, with the goal of illuminating the ethico-legal tensions at play and informing clearer regulatory and institutional guidelines.

**Methods:**

This study employed a qualitative, phenomenological design. Fourteen specialists were recruited via purposive and snowball sampling; semi-structured online interviews were conducted between January–May 2025 using a guide with four interrelated domains. Audio was transcribed verbatim and analyzed in MAXQDA-10. A three-stage, iterative thematic analysis (independent coding, expert consultation, framework refinement) was undertaken until thematic saturation was confirmed.

**Results:**

Thematic analysis identified four main themes: (1) legal knowledge and clinical approach to abortion, (2) moral reasoning in abortion decisions, (3) operationalizing conscientious objection in clinical settings, and (4) ethical conflicts between conscience and care. Each of these themes consisted of several subthemes.

**Conclusion:**

Abortion care in Türkiye is shaped by negotiations between law, morality, and institutional practice. Conscientious objection, though not legally recognized, is nonetheless practiced covertly as a contingent and relational stance, jeopardizing women’s reproductive autonomy. These findings underscore the fragility of reproductive rights and the urgent need for stronger policy frameworks to safeguard access, and autonomy.

**Supplementary Information:**

The online version contains supplementary material available at 10.1186/s12910-026-01380-z.

## Background

Voluntary, elective, or induced abortion refers to the intentional termination of a pregnancy within the legal parameters established by a given jurisdiction. In bioethical discourse, abortion has long been a contested practice because it raises questions about how to balance the interests, rights, and moral claims of the pregnant women with those attributed to the developing embryo or fetus. While some approaches center women’s autonomy, bodily integrity, and reproductive self-determination as the primary ethical considerations, others focus on the moral status of the embryo or fetus and the conditions under which it may warrant moral protection [21, 23, 35, 37, 39, 40, 43]. These competing perspectives have generated sustained philosophical debate and have informed divergent legal and policy frameworks across jurisdictions. For example, whereas many European countries permit abortion within until 12–24 weeks of gestation, often grounding access in considerations of women’s health and bodily autonomy, other contexts, particularly those influenced by religious or conservative moral traditions such as some Latin American countries, restrict or prohibit abortion by prioritizing moral status of the developing embryo or fetus [42].

Alongside theoretical and abstract debates over the women’s autonomy and moral status of the embryo/fetus, conscientious objection has emerged as a complex ethical issue in abortion practice. Conscientious objection in medicine refers to the right of clinicians “to refuse to perform tasks that would otherwise be regarded as part of their job” [31]. Although proponents argue that it protects clinicians’ moral integrity [6, 44], critics emphasize its potential to restrict women’s reproductive rights, reinforce gender inequality, obstruct access to safe care, and generate serious ethical problems and public health challenges in time-sensitive practices such as abortion [16, 26, 32, 33]. In line with the latter arguments, global evidence demonstrates that conscientious objection undermines abortion access both directly, through clinicians’ refusals, and indirectly, by reinforcing structural barriers [10]. When abortion services are denied, particularly in rural or resource-limited settings, women may be forced to continue unwanted pregnancies or resort to unsafe alternatives, thereby increasing risks of morbidity and mortality [45].

In Türkiye, abortion has been legally permitted since 1983 under the Law on Population Planning (No. 2827), which allows termination without the need to provide a justification up to the end of the tenth week of pregnancy [30]. Under this regulation, spousal consent is required if the woman is married, and in cases of medical necessity (such as fetal anomalies) or criminal circumstances, including rape, the legal time limit is extended to 20 weeks [34], pp. 110–111). Despite this permissive legal framework, access to abortion services in public healthcare institutions has been reported to decline over the past decade. Empirical studies indicate that healthcare providers frequently refuse to perform abortions on moral or religious grounds or cite institutional constraints [2, 19, 25, 29, 38]. As a result, many women are compelled to seek services in private healthcare settings, where access is less equitable and financial costs are significantly higher [29]. In some cases, women also rely on informal networks to obtain abortion-related information or support [15].

Türkiye, a majority-Muslim country with a secular constitution, offers a particularly instructive case for examining the gap between formal abortion law and clinical practice. For many years following its legalization, abortion services were widely available in public hospitals without the requirement to provide a reason up to the tenth week of pregnancy. However, since the early 2010 s, access to abortion in public healthcare institutions has been reported to decline,^1^ despite the continued legal permissibility of the procedure [28]. Women increasingly report being turned away from public hospitals with explanations such as “it is the doctor’s decision,” “Hospital management does not approve induced abortion,” or “Abortion is banned in public institutions” [2, 15, 28, 29]. These accounts point not to a change in law, but rather a growing disjunction between legal entitlement and clinical practice, shaped by broader political, institutional, and moral dynamics that warrant closer examination. Existing research highlights the role of pronatalist policies and reproductive governance mechanisms, such as increased surveillance of pregnancy and reproductive behavior, in constraining access to abortion services [3, 11, 20]. Together, these dynamics have produced a context in which abortion remains legally permitted yet increasingly difficult to access in practice, underscoring the need to examine how legal rights are mediated, reshaped, and at times undermined within healthcare institutions.

In clinical settings, refusals to provide abortion care seem to be more often rooted in moral objections held by individual clinicians or institutionally imposed constraints. This is particularly striking given that no legal provision explicitly grants healthcare professionals a right to conscientious objection in abortion care [18, 34, 41]. Article 18 of the Medical Deontology Regulation permits physicians to refuse care on “personal” or “professional” grounds, while Articles 24 and 28 address refusals arising from conflicts of interest or consultation requirements. In addition, Article 6 of the Occupational Health and Safety Law allows refusal only in situations involving violence or serious threats to personal safety. Crucially, none of these provisions authorize refusal on moral or ideological grounds, nor do they permit denial of care in emergency situations or where public duties are implicated. Nevertheless, the ambiguity regarding what “personal” might imply seems to facilitate the normalization of practices that function as de facto conscientious objection, despite lacking a clear legal foundation.

 The persistence of this discrepancy highlights a structural gap between formal legal rights and their realization in clinical practice, although abortion is legally available within specified limits: the effective exercise of reproductive rights becomes contingent on individual clinicians’ moral views or institutional cultures rather than on law. Understanding this gap requires closer attention to the lived experiences of OBGYNs, whose moral reasoning, institutional constraints, and professional vulnerabilities shape how abortion law is enacted in practice.

Yet, little is known about how OBGYNs in Türkiye understand, experience, and navigate induced abortion and conscientious objection in their daily practice. To our knowledge, no study has yet addressed this specific issue, even though it appears to be a critical factor in understanding the inaccessibility of abortion services in Türkiye. Existing scholarship largely approaches conscientious objection at a theoretical or normative level, without capturing how these tensions unfold in clinical contexts [2, 3, 28, 29]. Understanding clinicians’ perspectives is essential not only for developing ethically sound policies but also for ensuring that legal abortion rights are upheld without disregarding clinicians’ moral integrity.

This study carries a global significance as well because it confronts a core ethical challenge in reproductive healthcare: the erosion of women’s rights when legal protections are not matched by effective practice. Türkiye illustrates how reproductive rights, even when permissively regulated by law, can become unsustainable and inadequately protected within the health system and “in the shadow of reproductive governance” [3]. By documenting the perspectives of Turkish OBGYNs, the study offers one of the first empirical accounts of how perceived ambiguity on conscientious objection, combined with institutional pressures and individual moral reasoning, systematically restricts women’s access to legal abortion. These findings resonate globally, as similar patterns, where abortion is legally permitted yet effectively obstructed, are increasingly evident across Europe, Latin America, and beyond. By situating Türkiye, a secular yet majority-Muslim country, as an example where reproductive rights exist only on paper, this study underscores the fragility of reproductive rights and highlights the urgent need for stronger, ethically sound frameworks to safeguard women’s autonomy, equality, and access to care worldwide. Descriptively, it enriches international scholarship by providing a case study from a context where abortion rights are legally recognized yet remain precarious in practice.

The study is guided by the following research questions:

- What are clinicians’ views on induced abortion, and how do legal regulations on abortion influence their clinical practice?
- What are clinicians’ views on conscientious objection to abortion, and how do legal regulations (or their absence) affect its application in clinical practice?
- How do clinicians understand and respond to ethical challenges related to induced abortion, particularly when personal moral views intersect with professional responsibilities?

## Methods

### Design

This study was designed as a phenomenological qualitative inquiry. Qualitative research seeks to generate in-depth understanding by exploring participants’ experiences in relation to the research question, with participants selected purposively to capture relevant perspectives. Phenomenological design focuses on how individuals experience a given phenomenon, how they make meaning of those experiences, and how such meanings are shaped by context. Accordingly, analysis in phenomenological research attends to both participants’ lived experiences and the contextual conditions in which those experiences occur [7, 8].

### Sampling

The participant group consisted of specialist clinicians in Obstetrics and Gynecology. Purposive and snowball sampling techniques were employed in line with the aim of the study. Participation was voluntary, with no restrictions based on sociodemographic characteristics, years of professional experience, or institutional affiliation. Recruitment through snowball sampling began with one participant from Marmara University Hospital, which was readily accessible to the researchers, as is often the case in qualitative research. Each participant was then asked to recommend a colleague. In total, fourteen specialists agreed to take part, while others declined due to time constraints and some articulated their concerns about confidentiality in such a socially and politically sensitive issue.

Creswell [7] recommends a sample size of 6 to 18 participants for phenomenological research and advises continuing data collection until saturation is achieved. In this study, data saturation was assessed by examining whether new information emerged with each additional interview. The research team reached consensus that saturation had been achieved after the fourteenth interview. To confirm this, one additional interview was conducted, as no new themes emerged, it was not included in the final analysis. All interviews were anonymized, and no personally identifiable information was collected or disclosed.

### Characteristics of participants

Of the 14 participants, 11 are women and three are men. Their professional experience ranges from 1 to 15 years. Thirteen participants work in tertiary public hospitals across two institutions (six and seven participants, respectively), and one participant works in a secondary-level private university hospital. Additionally, two participants hold academic positions as full professors at a state university, while the remaining participants work as specialist clinicians in their respective institutions. All participants are employed in hospitals located in Istanbul.

Regarding their clinical practices, participants’ responses to the question “Have you ever performed an induced abortion during your professional career?” were as follows: three participants reported that they perform voluntary abortions up to the tenth week of pregnancy; five stated that they conscientiously object and would not perform abortions under any circumstances; and six indicated that, although they generally object, they would perform induced abortions when the woman has no other means of accessing abortion services.

### Data collection

In this study, data were collected through semi-structured interviews. The interview guide (see Supplementary File 1) was developed specifically for this research by MB, HBA, and KA, following a comprehensive review of the bioethics literature on conscientious objection, patient refusal in abortion care, and the moral concerns surrounding abortion practice. The guide included one demographic section followed by four interrelated main sections (comprising 15 short questions) exploring participants’ views and experiences regarding abortion, its legality, the moral status of the embryo, and conscientious objection. It was piloted with two medical residents to ensure clarity and comprehensibility and subsequently revised based on their feedback. Interviews were conducted online between January and May 2025, with participants’ informed consent, and were audio-recorded by MB, HBA, or KA on a rotating basis.

Interviews averaged approximately 35 min (range 28–42 min). Although this duration is shorter than is typical for phenomenological studies, it allowed participants to elaborate meaningfully within a shorter time frame because the interview guide was designed to elicit focused and in-depth reflections on four closely related domains. Two participants joined their interviews while waiting for patients in operating rooms, given their busy clinical schedule.

At the beginning of each session, participants were informed about the study, including assurances of confidentiality, the deletion of audio files after transcription, and their right to withdraw at any time without providing a reason. Informed consent was obtained prior to participation.

All interviews were anonymized, and no personal identifiers were recorded or shared. Each session began with a brief sociodemographic questionnaire, followed by the semi-structured interview guide. The study adhered to the principles of the Declaration of Helsinki and received ethics approval from the Marmara University School of Medicine (09.2024.1571) prior to data collection.

### Data analysis

All audio recordings were transcribed verbatim (AÇ, ANO, SDB, BP) and initially analyzed by three researchers (MB, HBA and KA) using MAXQDA-10 software. Based on established qualitative analysis methods [1, 7, 17, 36], a three-stage iterative thematic analysis was then conducted. In the first stage, two researchers (MB, HBA) independently conducted an initial holistic reading of the transcripts and coded the data according to their meanings and salient features, after which they reached consensus on the codes and preliminary themes. In the second stage, expert consultation (MS) was obtained to establish the analytical framework and evaluate it in relation to the research questions. In the third stage, two researchers (MB, HBA) re-analyzed the data in depth within this framework, refining and consolidating the themes. In the final verification stage, the themes were reviewed by the expert (MS), and the final analytical framework was confirmed.

## Results

### Qualitative findings

Four themes and ten subthemes were identified through thematic analysis: Legal knowledge and clinical approach to abortion (Interpretation of legal boundaries in abortion practice, Institutional and structural constraints, Clinical evaluation in abortion practice); Moral reasoning in abortion decisions (Divergent moral logics in abortion practice, Moral ontology of the embryo); Operationalizing conscientious objection in clinical settings (Legal and practical ambiguity of conscientious objection, Conflation of conscientious objection with other type of refusals, Managing patients under objection); and Ethical conflicts between conscience and care (Conscientious objection as a moral shield or burden, Contingency of conscientious objection). Themes and subthemes are represented in Table 1.

**Table 1:** Themes and Subthemes

Theme	Sub-themes
1.Legal knowledge and clinical approach to abortion	-Interpretation of legal boundaries in abortion practice -Institutional and structural constraints -Clinical evaluation in abortion practice
2.Moral reasoning in abortion decisions	-Divergent moral logics in abortion practice -Moral ontology of the embryo
3.Operationalizing conscientious objection in clinical settings	-Legal and practical ambiguity of conscientious objection -Conflation of conscientious objection with other type of refusals -Managing , patients under objection
4.Ethical conflicts between conscience and care	-Conscientious objection as a moral shield or burden -Contingency of conscientious objection

## Legal knowledge and clinical approach to abortion

Participants demonstrated a clear understanding of the legal framework governing abortion in Türkiye. They were familiar with the history of legalization both domestically and in other European countries and expressed broad agreement with the availability of legal and safe abortion services. This theme consisted of three sub-themes, which are described below.

### Interpretation of legal boundaries in abortion practice

Several participants stated that they perform abortions only within the legal limits, describing the law as the main reference point for their decision-making. They explained that the 10-week limit provides a clear framework that guides their actions and helps them feel secure in their professional role. For these clinicians, following the law was not experienced as a burden, but rather as a means to act with confidence and avoid uncertainty.

*“First of all, if it weren't legal, I wouldn't do it as a specialist. I would never get involved in something illegal, even if the patient wanted or needed it, even if it was for the patient's benefit.” (P9, F, 4 years).*

Several participants linked the legal limit to fetal developmental milestones and the safety of first-trimester procedures, noting that the timing reduces medical risks for the patient. For these clinicians, the 10-week limit was not just a legal requirement but also a practical guideline that aligned with their clinical experience.

*“A 10-week limit is reasonable because after 10 weeks, bone development and so forth begin in the fetus, making the procedure more complicated and, in this case, more risky for the patient. Therefore, the 10-week limit is a legal medical requirement.” (P 12, F, 1 yr).*

Most participants reported being satisfied with the current legal framework. A few participants, however, suggested that the regulations could be revised to make abortion available solely at the woman’s request. They explained that the current requirement for spousal consent in married women can delay the process and limit women’s ability to make decisions about their own bodies.

*“When we perform an abortion, we obtain consent from the mother and her husband. But it cannot be performed without the husband’s consent. Actually, it's the woman's body, the woman's decision, that's the dilemma. The child inside is also the husband's child, yes, but ultimately it's the woman's body. Perhaps the woman has had very risky pregnancies, threatening her life, but still, having that abortion is in the man's hands. I would like to change that. I would like it to be possible based solely on the woman's own statement.” (P2, F, 1 yr).*

Lastly, some participants mentioned that in cases of sexual abuse, the 20-week legal time limit for criminal cases can be difficult to meet because obtaining the required documentation and completing legal procedures may take several weeks. They suggested that the legal time frame should be extended in such situations to ensure that affected women can still access abortion services.

*“In abuse cases…it should be more flexible… weeks are lost getting documentation… For example, when I was an assistant, I delivered a 14-year-old girl's baby. Her uncle had raped her… She screamed, “Get the monster out of me!” as she gave birth. It was horrific.” (P1, F, 3 yrs).*

### Institutional and structural constraints

Participants reported that induced services have become increasingly difficult to access in public hospitals. They attributed this primarily to systemic factors rather than to individual conscientious objection. The most commonly cited barriers included the prioritization of emergent cases, limited operating room availability, insufficient anesthesia support, and administrative restrictions. Several participants explained that, because of these institutional and structural constraints, patients are often referred to other hospitals or private facilities. Clinicians working in tertiary care centers added that abortions are frequently deprioritized in hospital scheduling, as they are expected to focus on urgent or complex procedures.

*“It is not practiced because it is necessary to perform it under general anesthesia, as patients may be affected by pain. Because we are a tertiary care center, in the room where you will perform general anesthesia, there is not much time left for such elective procedures, as there are mostly surgeries for cancer or other critical indications. Legally, the patient has the right to ask for it, but we politely tell them that this cannot be done due to technical conditions.” (P8, M, 15 yrs).*

Some participants reported that, even though they personally had no objection to abortion, their hospitals did not provide the service. They explained that this was due to informal or unwritten institutional policies and managerial decisions that effectively prevented abortions from being performed, despite the procedure being legally permitted.

*“Even though it was clearly and explicitly stated in the Population Planning Law, some administrators said that abortions would not be performed. As a result, although abortion was legally permitted, we were told not to provide the service in the clinics where I worked… so we told patients that we did not perform it in the clinics. This decision usually came from the clinic director or the head physician. So, in practice, the procedure was not carried out in our hospital. You could describe it as an informal or unwritten sanction, there was no legal basis for it, but it was enforced nonetheless. In such cases, I referred patients to doctors I knew and trusted in private hospitals.” (P9, F, 4 years).*

### Clinical evaluation in abortion practice

Participants explained that they evaluate several factors before performing an abortion, rather than approving every request automatically. They mentioned considering medical safety, the patient’s previous obstetric history, and whether the decision was made voluntarily as key elements of their assessment.

*“Is the case suitable for abortion? I am evaluating any presence of cesarean scar, bleeding diathesis, psychiatric problems… and the risk of complications are on my mind.” (P9, F, 4 yrs).*

In addition to assessing clinical risks, several participants said they ask patients about the number of previous pregnancies, contraceptive use, and reasons for seeking an abortion. Some acknowledged that these questions are not strictly required but explained that they ask them as part of their routine practice.

“We ask… How many children? Protected? Why terminate? We shouldn’t ask, but feel responsible.” (P3, F, 1 yr)

*“Ethically speaking, if a patient considers abortion a method of contraception and comes with that expectation, I do feel uncomfortable performing the procedure. However, if the patient is married, the pregnancy occurred despite using contraception, and it is unintended, then I do not see a dilemma. But if, as I mentioned, the patient had unprotected sex, is generally careless, and has done this multiple times, or is likely to do so again, that creates a different situation…” (P14, F, 1 yr).*

Some participants suggested that policies could be introduced to prevent repeated abortions, including requiring contraception, such as IUD insertion, after the procedure. They explained that such measures could help reduce the number of repeat terminations they encounter in practice.

*“A compulsory contraception method like IUD should be applied after abortion… It’s not right to repeatedly terminate pregnancies without protection.” (P3, F, 1 yr)*

## Moral reasoning in abortion decisions

Participants described a variety of approaches to responding to patients’ abortion requests, reflecting different understandings of their role as clinicians. Some reported performing abortions within the legal limits while leaving the decision entirely to the patient, whereas others said they engaged patients in discussion to confirm certainty or address potential regret. A smaller group stated that they do not perform induced abortions unless medically necessary, citing religious reasons and their views on the moral status of the embryo. Taken together, these accounts show that abortion care is approached in diverse ways, ranging from full deference to patient choice to refusal based on personal belief, which are organized into two subthemes below.

### Divergent moral logics in abortion practice

Some participants performed abortions within legal limits, fully deferring the decision to the patient and deliberately separating personal or religious beliefs from professional action as long as legal criteria were met:

*“If a woman decides that she does not want to continue the pregnancy, because she is not ready, not prepared emotionally or financially, or feels unable to care for a child, then forcing her to give birth can have serious consequences. The child may be born into a situation where they are not adequately cared for or loved, and in some cases may even be placed in institutional care. At the same time, the woman herself may experience significant psychological distress as a result of carrying and raising a child she did not want. In some cases, this can lead to long-term emotional harm or neglect of the child. For these reasons, I believe that the decision should ultimately belong to the woman. She should have the right to decide whether or not to continue the pregnancy.” (P2, F, 1 yr).*

*“I completely defer to the patient’s opinion… if the patient has made this decision, I can only carry it out… My religious views are irrelevant.” (P4, F, 2 yrs)*

Others provided abortion services but adopted a more conditional stance, emphasizing their role not only as service providers but also as professionals with responsibilities toward patients’ decision-making. These clinicians described discussing potential issues such as regret, coercion, or patterns of repeated unprotected pregnancies, and considered it part of their duty to address such concerns with patients.

*“Sometimes a woman says, ‘I don’t feel ready for a pregnancy; I’ve only been married for a year,’ and in those cases I feel the need to explain things more carefully. Since I specialize in reproductive endocrinology and infertility, I tell patients that while this pregnancy may feel unexpected or overwhelming now, there are many people who struggle for years to conceive. I ask them to consider whether they might later regret ending the pregnancy, especially if they later wish to have a child and face difficulties. This is not like taking a painkiller to relieve a headache, it’s irreversible. That’s why I believe it’s important to think carefully before making such a decision.” (P7, F, 14 yrs).*

*“I’m not influenced by religious beliefs, but when a patient is irresponsible, for example, when she has little knowledge about contraception and treats abortion as something easily repeatable, thinking, ‘I can always get an abortion if I get pregnant’, that really troubles me…I once performed an abortion for a friend, for example. I asked what method of contraception they were using, and they said the morning-after pill. That is not an appropriate method of regular contraception. We usually recommend condoms, birth control pills, or longer-term methods such as IUDs or implants” (P14, F, 1 yr).*

Finally, some declined to perform induced abortions altogether, citing personal or religious beliefs. They emphasized that they only performed abortions in cases of medical necessity, framing their refusal as an exercise of legally recognized conscientious objection:

*“I don't perform elective abortions; we have the right to conscientious objection. I perform abortions only when medically necessary since there is a medical condition involved, there is no problem.. but I personally do not perform elective abortions… that’s because of my beliefs.” (P13, F, 5 yrs).*

*“I tell them, ‘My colleagues may perform it, but I do not.’ I don’t perform elective abortions because of my beliefs. The only abortions I perform are in cases where the fetus’s heart has already stopped or when there has been a miscarriage and tissue remains inside. In those situations, the procedure is medically necessary, and that is when I intervene.’” (P11, F, 1 yr).*

### Moral ontology of the embryo

Most participants described the embryo as a biological entity beginning at fertilization. They frequently referred to biological indicators, such as the heartbeat or the development of a circulatory system, as important markers of life. These features were often cited as reference points for understanding when life begins, without necessarily distinguishing between biological activity and moral significance.

*“From the zygote stage onward, it is a particle of power with the potential to form a human being… if we leave it to its own, nine months later we will have a human being.” (P6, F, 6 yrs).*

*“The fertilized egg… is also a living being… with a chance to live if you give permission. That’s why I think both [woman and the embryo] have roughly equal rights.” (P7, F, 14 yrs).*

*“Seeing the heartbeat is sufficient to conclude that life has begun.” (P13, F, 5 yrs)*

Other participants challenged the idea that moral status begins at fertilization, instead advocating a gradual and emergent attribution of moral status. They pointed to milestones such as implantation, organ development, viability, or birth as thresholds that confer increasing moral significance.

*“It has a primitive circulatory system… like a plant, it is alive, but that doesn’t mean it is a person.” (P8, M, 15 yrs)*

*“We can evaluate it as a human being after it is born… I keep it in a gray area.” (P11, F, 1 year)*

*“Now, before 20 weeks it’s a miscarriage, after that it’s a birth… so I’d say 20 weeks is when it acquires moral significance.” (P2, F, 1 yr)*

Some participants referred to both religious teachings and individual rights when describing the moral status of the embryo. They mentioned that Islam regards embryonic life as valuable, while also noting that decisions about abortion ultimately depend on the woman’s choice, especially in the early stages of pregnancy. These participants described the embryo as important but still dependent on the pregnant woman, whose decision they considered central to the outcome.

*“Islam sees every stage of life as valuable… but until the fetus can survive independently, the woman’s decision is valid.” (P7, F, 14 yrs)*

## Operationalizing conscientious objection in clinical settings

Participants described a range of perspectives on how conscientious objection is understood and applied in clinical practice. In the absence of clear legal regulation, many highlighted the legal ambiguity of conscientious objection, noting uncertainty about whether refusals were formally protected or regulated. Others tended to conflate conscientious objection with other types of refusals, extending the concept beyond conscience-based objections to include refusals related to patient behavior, communication problems, or personal discomfort. All participants emphasized the practical aspects of managing patients under objection, describing strategies such as referral, counseling, or redirecting patients to alternative providers. These perspectives are presented in three subthemes: legal and practical ambiguity, conflation with other refusals, and management of patients under objection.

### Legal and practical ambiguity of conscientious objection

Participants often expressed ambiguous understandings of the legal status of conscientious objection. Many were uncertain whether it is formally recognized in Türkiye, particularly beyond the context of abortion. Some considered it as a professional discretion rather than a clearly defined legal entitlement.

*“Ethically, a doctor has no right to refuse patients [in emergencies]… But if there is no such risk, I think they can refuse. Still, we usually refer them.” (P6, F, 6 years)*

*“There is no such legally defined provision but healthcare workers have the right to refuse treatment or care under certain conditions… In my opinion, except for emergency cases, there needs to be more flexibility in this issue.” (P8, M, 15 years).*

*“I don't know the legal aspect of this. I think it's a commonly used definition, but I'm not sure if it's specific to abortion. I don't think so.” (P12, F, 1 year)*

### Conflation of conscientious objection with other types of patient refusals

Several participants frequently cited disrespect, communication breakdowns, trust issues or prior negative engagements as valid reasons to refuse patients. These were often discussed as to what would formally qualify as conscientious objection. While such refusals may be ethically justified in some cases, they blur the distinction between conscientious objection and other forms of refusal not based on moral or religious conviction.

*“If the patient is disrespectful… or if there is verbal violence…If there is a problem in communication with the doctor, I think the doctor should have the right to refuse.” (P3, F, 1 year).*

*“I think no doctor is actually obligated to do anything. Because they may not have the ability to do it, or they may not want to do it.” (P2, F, 1 year)*

*“The patient can say, ‘I don’t want to be examined by this doctor,’… Similarly, if a doctor has difficulty communicating with a patient, if not an emergency, the right to refuse should exist.” (P7, F, 14 years).*

*“If a doctor feels the patient doesn’t trust them, I think that alone can be enough to refuse the patient.” (P5, M, 2 years).*

### Managing patients under objection

Most participants reported that when they declined to perform an abortion, whether for personal, institutional, or situational reasons, they still wanted to make sure that patients received care via referral. Referral was described as the primary way of managing such situations, often involving practical steps to guide patients to another provider or facility. For some, referral had become a routine part of practice, serving as a form of soft objection when they could not, or chose not to, perform the procedure themselves.

*“I refused to perform elective abortion on moral grounds… but perhaps in the patient’s final days… this right could be exercised without taking it away from the patient… The patient's legal right also comes into play here. That is, the patient also has the right to terminate the pregnancy. I refused this on moral grounds, but perhaps in the patient's final days, she has this right. This can be done without taking that right away from the patient, that is, without taking a right away from the patient, without hindering the patient… perhaps by referring them to someone else.” (P3, F, 1 yr).*

*“I believe conscientious objection is possible, but it must not result in harm to the patient or obstruct access to care. I may refuse to perform an abortion on moral grounds, but I ensure that the patient can still receive the service somehow. If another provider is available, then there is no problem. However, if no one else is willing to perform the procedure, this is no longer a matter of conscientious objection but rather an obstruction of care. That is unacceptable. The system must be organized in a way that guarantees the service is provided. It should not matter who performs it, as long as the patient receives the care she needs.” (P2, F, 1 yr).*

Some participants suggested structural solutions to make access more reliable, such as appointing state-supported providers in each region to whom patients could be referred when conscientious objection is exercised.

*“If the doctor is given this right, the state should offer an alternative… not everyone will do everything. The state should have doctors [who perform abortions] in certain regions… patients should be referred through the system.” (P12, F, 1 yr).*

Others pointed out that abortion refusal is rarely declared explicitly, especially in public hospitals, where clinicians did not voice a direct moral refusal but instead referred to institutional constraints, for example, stating that “it’s not done here”, as a way of declining abortion requests without personal disclosure. This not only creates informational opacity for patients but also raises ethical concerns about the fairness and transparency of care delivery.

*“It’s a bit difficult to say directly that I don’t do it… Since the institution I work for is a busy hospital, it’s easy to use that as an excuse… But if the patient asks me directly whether I perform it, I would say, ‘No, I don’t.’ In this institution, for example, there are those who say they will do it, those who say they won’t, those who say they will do it for financial reasons, those who say they won’t for religious reasons, and those who refuse for moral reasons.” (P8, M, 15 yrs).*

*“It usually goes like this: there are doctors who do it and doctors who don't. Patients find doctors who do it… In state hospitals, it’s not something done routinely, so we refer them.” (P11, F, 1 year).*

Even when clinicians stood firm on their objection, many emphasized the importance of nonjudgmental communication, timely information, and proactive referral to reduce distress. They described making efforts to inform patients about their legal rights and direct them to where the procedure could be performed.

*“I draw that line clearly that I do perform abortions… I refer them and give them the necessary information, and that they have up to 10 weeks to get it done…. I believe I approach it professionally. For example, when a patient comes to me, I explain everything in detail. I tell them that the procedure is legal up to 10 weeks, that after 10 weeks it is not permitted, and how it is performed under anesthesia. I am straightforward and say that it may be easier to access the service in a private hospital. I share all the information I have, even when it concerns something I personally do not support. I do not withhold information; I try to provide the patient with everything they need to know*.*” (P11, F, 1 year).*

Finally, some participants highlighted the emotional toll and potential misunderstandings that can arise when refusal is poorly communicated or not accompanied by adequate explanation.

*“I have had patients who came seeking an abortion, and I provided them with the necessary information without any difficulty. However, when adequate information is not provided, patients can become distressed, often because they are encountering the situation for the first time, feel uncertain about what to do, and have made a sudden decision. When the person they are speaking to then says they cannot help, the situation can quickly become tense.” (P6, F, 6 years).*

## Ethical conflicts between conscience and care

Participants who reported applying conscientious objection in abortion cases described experiencing tensions between their own moral convictions and their professional responsibilities toward patients. Rather than presenting their stance as absolute, most emphasized the challenges of balancing personal beliefs with the time-sensitive and emotionally charged context of abortion care. Their accounts highlighted efforts to minimize harm, preserve trust in the clinician–patient relationship, and respect women’s autonomy while managing their own moral concerns. These ethical conflicts were expressed in different ways, reflected in two subthemes: conscientious objection as a moral shield or burden and the contingency of objection.

### Conscientious objection as a moral shield or burden

Participants frequently described conscientious objection as important for preserving their moral integrity and ability to continue practicing medicine with motivation. Several stated that being compelled to act against their beliefs would undermine their peace of mind and emotional well-being, influencing their professional performance.

*“There should be a right to conscientious objection because otherwise, doctors cannot practice their profession with peace of mind. After all, a doctor is not a robot but a human being with their own ethical values, in my opinion.” (P8, M, 15 yrs).*

*“What kind of positive effects can a legal right to conscientious objection have? …I want to be able to sleep peacefully when I go to bed. Therefore, peace of mind is actually what allows you to continue to love your profession." (P7, F, 14 yrs).*

*“Being a doctor is not something that can be done without passion… The right to conscientious objection allows me to embrace my profession.” (P11, F, 1 yr)*

Some participants recalled situations where they felt pressured to perform procedures they would otherwise object to, which they described as emotionally draining and morally burdening.

*“If someone forces me to do it, I won't feel at ease with my conscience when I do it… A person's choices are none of our business… I just don't want to be involved in something I don't want to do.” (P7, F, 14 yrs).*

A few participants noted that in regions where abortion services are already limited, the exercise of conscientious objection can harm patients, framing objection to abortion itself as a moral burden. In these settings, objection was described not only as a way to protect clinicians’ moral integrity but also as a practice that further complicates patients’ access to timely care.

*“It’s not like a routine pregnancy check-up… it is time sensitive… If I refuse her, she is somewhat deprived of those rights, of course…” (P11, F, 1 year)*

*“If the doctor doesn't take on this [social] responsibility, then something else, like unsafe abortion or harm to patients, will happen… we doctors must act with the social structure in mind.” (P12, F, 1 yr).*

### Contingency of objection

Several participants acknowledged the ethical tension involved, especially when the patient has no other alternatives. They also noted that their refusal was not absolute, but rather depended on the circumstances. They reported being willing to reconsider and compromise their decision in urgent cases or if no other option was available, in order to prevent patient harm.

*“Yes, I refuse on moral grounds, but… if she has no choice, it [my decision to object] will be reconsidered and the patient will not be harmed. If there is no one else to do it, then I must do it. That is where the line is drawn. If someone else can do it, then I may not have to. But if no one else can do it, then this is no longer a right to refuse; it is completely withholding service, taking away someone's right to care. And that is not the job of a healthcare worker. Healthcare workers provide care. That's why the line must be drawn clearly. So conscientious objection can be used in a way that doesn't prevent someone from accessing the service, but if it completely prevents the service, that's no longer a conscientious objection.” (P2, F, 1 yr).*

*“I am conscientiously opposed to abortion…But she has no other choice?… Of course, it's easy to say that I would do it as I am now, but I would say that I would do it this way or that way in such a scenario. If we change it to something more meaningful to me, let's say the patient's husband slapped me five minutes ago and I have to deliver his wife's baby, would I do it? Again, I think I would do it because that is what the patient needs and she has no other choice. So, I wanted to elaborate a bit by giving this example because it evokes a stronger feeling in me that I shouldn't do it… Generally, I try to solve the patient’s problem. I try to be the final stop.” (P9, F, 4 years).*

Several participants acknowledged the difficulty of maintaining an objective decision in time-sensitive cases, especially when patients were approaching the 10-week legal limit. In these cases, some chose to provide abortion care to prevent the patient from losing access.

*“Nine weeks and five or six days…There are one or two days left. If you don't take care of the patient's request… their [legal] rights [to abortion] will be lost legally… So in this case, I would perform abortion.” (P1, F, 3 years).*

## Discussion

This study provides accounts of how OBGYNs in Türkiye understand, experience and navigate induced abortion and conscientious objection in everyday clinical practice. While abortion is legally permitted till the end of the 10th week of pregnancy, our findings reveal that clinical decision-making and accessibility of abortion services are determined by much more than the law. Participants’ accounts highlight the interplay of legal frameworks, moral reasoning, institutional constraints, personal conscience and religious beliefs, each contributing to the practical realities of abortion provision in public healthcare institutions. Rather than treating these domains as separate, clinicians experience them as overlapping and, at times, conflicting dimensions that configure their ethical and professional responsibilities toward both patients and themselves.

Within the scope of our first research question, our findings indicate that the law functions for clinicians not merely as an external regulatory boundary but as an internal moral framework through which they interpret and justify their practice. The 10-week limit was described less as an abstract number than as a source of certainty, offering reassurance that their actions were legally permissible. It informs the ethical space in which decisions are made and ultimately structures the conditions under which patients gain—or are denied—access to care. Comparable dynamics have been observed in Brazil and Poland, where restrictive legal frameworks operate not only as external constraints but also as integral elements of clinicians’ moral reasoning, shaping how they justify their actions and interpret their professional responsibilities [13].

Furthermore, several participants regarded 10-weeks not only as a legal limit but also as a clinically safe boundary, citing increased risks beyond it, although medical evidence does not support this claim of a sharp rise in risk at exactly 10 weeks. The WHO [46] emphasizes that abortion remains very safe when performed with recommended methods by trained providers even beyond 10 weeks, though procedures may take longer and carry slightly increased risks of complications such as bleeding or infection. These risks increase gradually rather than abruptly, meaning the 10-week mark does not represent the clinically safest deadline. Participants nevertheless appeared to align their clinical reasoning with the law, effectively internalizing the statutory limit as if it were also a clinical threshold. In this way, the law became part of their moral landscape, providing clarity in the midst of a morally and socially contested practice.

At the same time, participants emphasized that legality does not directly translate into access. Their narratives highlighted the weight of systemic conditions, such as prioritization of emergent cases and thus lack of operating room availability, as well as informal institutional policies, which often compelled them to refer patients elsewhere. In these accounts, law appeared as a necessary but insufficient condition for access: abortion care was influenced not only by statutes but also by the hidden infrastructures and bureaucratic logics of the healthcare system. This gap between legal entitlement and clinical reality resonates with broader concerns in the literature about the erosion of abortion provision in public hospitals in Türkiye, despite its continued legality [2, 3, 22, 29].

Regarding our second research question, our findings indicate that clinicians did not experience conscientious objection as a fixed or uniform category. In the absence of explicit legal regulation in Türkiye, conscientious objection was experienced less as a clearly defined right and more as a matter of personal interpretation and institutional habit. For instance, participants noted that objection was often enacted covertly, particularly in public hospitals, through appeals to institutional barriers (e.g., “it’s not done here”) rather than explicit moral refusal, a pattern also reported in the literature [2, 29]. While this approach spared clinicians from direct confrontation with patients, it created opacity for patients and raised concerns about transparency and fairness in care delivery [3].

Moreover, the boundaries of conscientious objection were blurred by participants’ accounts that extended refusal beyond matters of conscience to include issues such as disrespectful patient behavior, communication breakdowns, or lack of trust. In the absence of clear regulation, conscientious objection becomes conflated with these other forms of non-provision, allowing clinicians to present a wide range of refusals as morally legitimate. Clinicians described operating within "gray zones" of practice where the boundaries between professional duty, institutional expectations, and personal conscience were unclear.

At this point, it may be tempting to argue that conscientious objection in Türkiye should be regulated in clear legal terms. Unlike many European countries and the United States, there is currently no explicitly recognized right to conscientious objection against a medical service in Türkiye. However, it is unclear whether introducing a formal legal right to conscientious objection would ultimately benefit or harm patients in this context. A comparative study of several countries found that poorly regulated conscientious objection significantly reduced the number of providers willing to perform abortions, thereby limiting patient access even when abortion remained legal [5]. Similarly, a study from South Africa demonstrates that the absence of clear legal and clinical guidelines has led many clinicians to rely on informal practices and personal judgment when invoking conscientious objection in abortion care [24]. This suggests that both not regulating conscientious objection can create distinct challenges for ensuring equitable access to abortion care.

On the other hand, legal protection of conscientious objection might also increase the number of clinicians invoking objection, potentially leaving women in situations of desperation. Evidence from literature shows that widespread conscientious objection can lead patients to seek costly private alternatives, thereby exacerbating inequality [12]. Evidence from Italy demonstrates that widespread legal protection of conscientious objection can result in over 70% of gynecologists refusing to perform abortions, producing severe geographic disparities and delays [4]. Similarly, in Poland, legal recognition of conscientious objection has enabled near-complete institutional refusal in some regions, effectively nullifying abortion access despite formal legality [14]. Thus, the impact of regulating conscientious objection may vary depending on the context. 

From an ethical perspective, in Türkiye where abortion services are already unevenly accessible [29], formally recognizing conscientious objection carries significant risks. As our findings suggest, even in the absence of explicit legal protection, refusals are already widespread and often institutionally normalized. Introducing an explicit legal right to conscientious objection may therefore further legitimize refusals, increase their prevalence, and contribute to the consolidation of abortion services within a limited number of institutions or the private sector, thereby deepening existing inequalities in access.

At the same time, the current situation, characterized by legal ambiguity and the interpretive space it creates, also poses serious challenges. While this ambiguity may occasionally allow room for negotiation or informal accommodation, it more often carries the risk of inconsistent practices, institutional discretion, and uncertainty for patients. In this sense, the problem is not simply the absence or presence of regulation, but how regulatory frameworks are designed and enforced. Rather than formalizing conscientious objection as an individual entitlement, a more ethically defensible approach may lie in developing policies that prioritize continuity of care, institutional responsibility, and guaranteed access, such as requiring public hospitals to ensure the availability of abortion services regardless of individual objections. Such an approach would address the harms associated with both unregulated conscientious objections and institutional non-provision, while avoiding the normalization of conscientious objection as a mechanism that undermines access to care.

Regarding our final research question, our findings suggest that clinicians rarely described objection as absolute. Instead, they portrayed it as contingent, an ongoing negotiation shaped by legal ambiguity, institutional constraints, and relational dynamics with patients. In phenomenological terms, objection did not emerge as a fixed stance but rather as part of clinicians’ lived negotiation of care and conscience. Within this negotiation, the boundaries of law, morality, and responsibility were continually reinterpreted in the practice. Rather than simply *applying* a principle, clinicians described inhabiting a space of tension where the intersections of law, morality, and professional duty were constantly renegotiated. This lived dimension of objection illustrates that conscience is not exercised in isolation or within a vacuum, but rather emerges through encounters with patients, colleagues, and institutional norms. Thus, the meaning of conscientious objection is constituted in practice according to patients’ realities. From a phenomenological perspective, conscientious objection seems less an act of withdrawing from care than a mode of engaging with its moral complexity, an interpretive practice in which clinicians seek to reconcile their sense of moral integrity with the needs and expectations of others. Similar attitudes toward objection have been reported in other countries. For instance, Norwegian GPs describe objection as negotiated, relational, and intertwined by institutional and legal limits rather than absolute [27]. Likewise, U.S. clinicians have described objection as contingent, often mediated through patient relationships and institutional constraints [9].

At this point, it is important to note that although some clinicians may be motivated by a sense of moral obligation to provide abortion care, a stance sometimes referred to as conscientious commitment or conscientious provision, none of the participants in this study explicitly articulated such a position during the interviews. The absence of explicit references to conscientious commitment may reflect the particular composition of the sample or the broader professional and institutional context in which such views are expressed. Importantly, this absence should not be interpreted as evidence that conscientious commitment does not exist in practice, but rather as a reflection of the limitations of the data and the specific conditions under which the interviews were conducted.

### Limitations of the research

This study has several limitations that should be acknowledged. First, the findings of this study are necessarily shaped by the experiences of the participants and by the specific contexts in which those experiences occurred, as is characteristic of qualitative phenomenological research. Consequently, experiences of the same phenomenon may differ when examined among other participants or within different contexts, such as varying institutional, socio-cultural, or regional settings. Second, as with all qualitative research, the findings reflect clinicians’ reported experiences and interpretations rather than direct observation of clinical encounters. Lastly, the sensitive nature of abortion and conscientious objection may have influenced how openly participants shared their views, despite assurances of confidentiality.

## Conclusions

Taken together, the findings of this study show that abortion care in Türkiye is shaped by a complex negotiation between law, morality, and institutional practice. Although OBGYNs often rely on the 10-week legal limit for clarity and protection, legality alone does not guarantee access for patients. Instead, decisions are filtered through personal moral reasoning, institutional culture, and the ambiguities of conscientious objection, which is frequently enacted without clear legal guidance. Clinicians’ accounts portray objection not as a fixed stance but as a contingent and relational practice: sometimes protective, sometimes burdensome, and often mediated through referral.

Our findings suggest that clinicians, even within a relatively small sample, already feel supported in refusing to provide induced abortion services despite the absence of explicit legal protection. Formalizing such a right would likely entrench existing barriers, accelerate the shift of induced abortion provision toward the private sector, and deepen socioeconomic inequalities in access to care. Although the current legal ambiguity is far from ideal, it nonetheless leaves some room for negotiation, referral, and case-by-case flexibility. Importantly, our findings also contribute to broader international debates on conscientious objection by demonstrating that, even in the absence of formal legal recognition, conscientious refusal can become normalized in practice and significantly shape clinical realities. Regulating conscientious objection as a formal right risks further legitimizing these dynamics and may ultimately exacerbate restrictions on access to abortion care.

In light of our findings, strengthening policy and institutional frameworks alone is insufficient without parallel efforts to address the ethical, educational, and professional dimensions of abortion care. Beyond ensuring formal legal access, there is a need for targeted interventions that support clinicians in navigating the ethical and practical complexities of abortion provision. This includes structured education on the medical risks of unsafe abortions, clear guidance on the appropriate use of pain management and anesthesia, and an emphasis on timely care given the legal limit of induced abortion. Equally important is fostering professional reflection on the scope and limits of conscientious objection, including its distinction from other forms of refusal and its implications for patients’ access to care. Finally, training that encourages critical reflection on stigma, moral judgment, and gendered assumptions, such as those surrounding repeat abortions, may help cultivate a more ethically grounded and patient-centered clinical practice. Together, these measures can contribute to a more coherent and ethically robust framework for safeguarding access to lawful abortion care in Türkiye.

## Supplementary Information

Supplementary Material 1.

## Data Availability

The datasets generated and/or analyzed during the current study are not publicly available due to confidentiality and ethical considerations, in accordance with the principles of the Declaration of Helsinki. Participants were assured that all recordings would be deleted after transcription and would not be shared with third parties. Given the politically and professionally sensitive nature of abortion and conscientious objection in Türkiye, sharing the data—even in transcript form—could compromise participant anonymity and trust. However, data are available from the corresponding author upon reasonable request. The primary author (MB) affirms that all analyses presented in the manuscript are based on securely stored, anonymized transcripts used solely for research purposes in line with approved ethical standards.

## Footnotes

1 While abortion refusals in healthcare institutions are sometimes attributed to resource limitations, no empirical study to date has identified resource scarcity as the primary cause of restricted access in Türkiye.
