# Supplementary material for: Ethical perspectives of obstetricians and gynecologists on induced abortion and conscientious objection in Türkiye: a phenomenological study
Source: BMC Med Ethics. 2026 Feb 26;27:81. doi: 10.1186/s12910-026-01380-z (PMC13097852; doi:10.1186/s12910-026-01380-z)
Supplement: Supplementary file 1 — Supplementary Material 1. [file 12910_2026_1380_MOESM1_ESM.docx]

**Socio-Demographic Information**

- What is your gender? (Female / Male)
- What is your marital status?
- What is your birth year?
- What year of your specialization are you currently in?
- What type of institution do you work in? (State hospital / Private hospital / University hospital / Private clinic)
- How would you describe your level of religiosity/ religious belief? (On a scale from 1 to 5)

**Section I: Ethical and Legal Awareness Regarding Abortion**

1. What is voluntary (induced) abortion, and under what circumstances is it performed?
2. How is voluntary abortion carried out as a medical procedure?
3. Could you describe its legal status in Türkiye? (When, under what conditions, and by whom can it be performed?)
4. How do you think legal regulations concerning abortion in Türkiye should be defined or limited?
5. What factors influence your decision-making regarding abortion? (e.g., ethical considerations, professional responsibility, religious beliefs)
6. Do you ever find yourself in ethical conflict when performing abortions?

**Section II: Ethical Views on the Embryo and Fetus**

1. When do you believe human life begins?
2. From what point do you think the embryo acquires human qualities?
3. What are your views on the moral status/significance of the embryo?

**Section III: Ethical and Legal Awareness Regarding Conscientious Objection**

1. What does conscientious objection mean to you, and what does it encompass?
2. Have you ever exercised conscientious objection in a clinical setting?
3. What do you think are the possible positive and negative effects of conscientious objection in clinical practice?

**Section IV: Views on Conscientious Objection and Abortion**

1. Have you ever performed a voluntary abortion during your professional career?
2. If abortion were within legal limits but conflicted with your personal beliefs or ethical values, would you exercise your right to conscientious objection?
3. How do you approach a patient requesting a voluntary abortion?
4. If you were working in a healthcare facility where you are the only accessible doctor (for example, in a district where referring the patient elsewhere would be difficult) and a patient requested an abortion that you personally object to, how would you proceed?
